# Retraction Note: A compact photometer based on metal-waveguide-capillary: application to detecting glucose of nanomolar concentration

**DOI:** 10.1038/s41598-021-91478-8

**Published:** 2021-06-02

**Authors:** Min Bai, Hui Huang, Jian Hao, Ji Zhang, Haibo Wu, Bo Qu

**Affiliations:** 1 Department of Electronic Science and Technology, Faculty of Electronic Information and Electrical Engineering, Dalian University of Technology, Dalian, 116024 China; 2 Jordan Valley Semiconductors UK Ltd, Shanghai, 201206 China

Retraction of: *Scientific Reports* 10.1038/srep10476, published online 28 May 2015

The authors have retracted this article.

In the study described in our article, the metal-waveguide-capillary (MWC)-based photometer was applied to two samples: the red-ink solution (RIS) and the glucose solution. However, we recently found that the detection results with the RIS cannot be reproduced. This may be due to contamination of the RIS, as the reagent bottles used for the RIS measurement were not clean. In light of this, the performance metrics and optical-path enhancement reported are incorrect, as well as the suggested mechanism of light scatter.

All authors agree to this retraction.

